# Supplementary material for: Relationship between People’s Interest in Medication Adherence, Health Literacy, and Self-Care: An Infodemiological Analysis in the Pre- and Post-COVID-19 Era
Source: J Pers Med. 2023 Jul 1;13(7):1090. doi: 10.3390/jpm13071090 (PMC10381156; doi:10.3390/jpm13071090)
Supplement: Supplementary file 1 [file jpm-13-01090-s001.zip › jpm-2400110-supplementary.pdf]

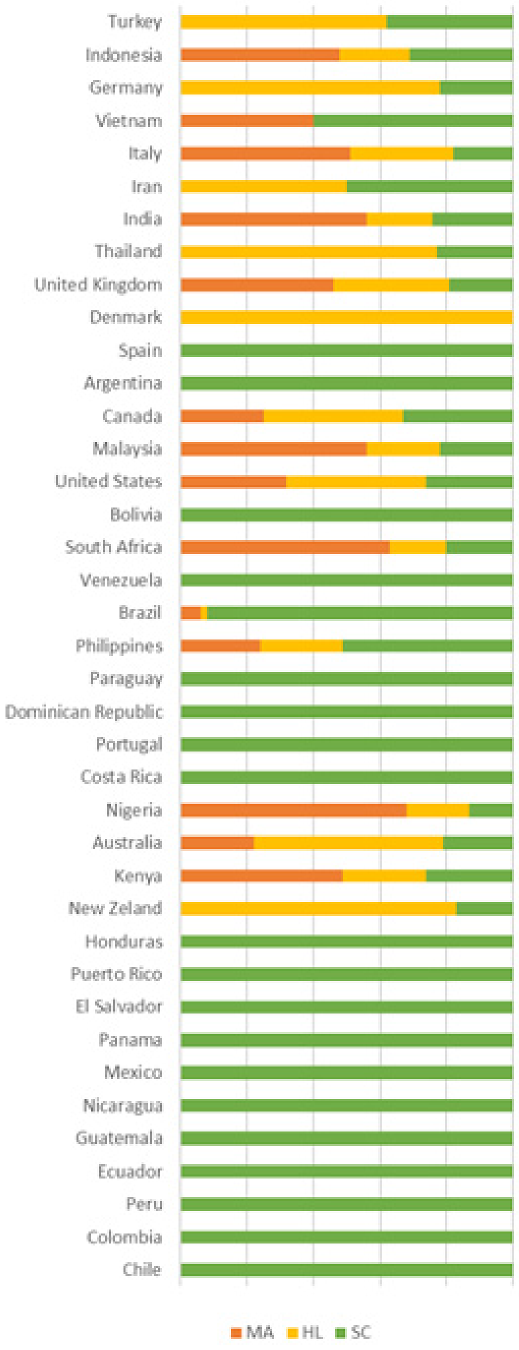

Supplementary Figure 1. Comparison of RSVs between different countries

Abbreviations: HL, health literacy; MA, medication adherence; SC, self-care
